# L-Amino Acid Oxidases From Mushrooms Show Antibacterial Activity Against the Phytopathogen *Ralstonia solanacearum*

**DOI:** 10.3389/fmicb.2020.00977

**Published:** 2020-05-19

**Authors:** Jerica Sabotič, Jože Brzin, Jana Erjavec, Tanja Dreo, Magda Tušek Žnidarič, Maja Ravnikar, Janko Kos

**Affiliations:** ^1^Department of Biotechnology, Jožef Stefan Institute, Ljubljana, Slovenia; ^2^Department of Biotechnology and Systems Biology, National Institute of Biology, Ljubljana, Slovenia; ^3^Faculty of Pharmacy, University of Ljubljana, Ljubljana, Slovenia

**Keywords:** bacterial wilt, antimicrobial, *Amanita phalloides*, *Clitocybe geotropa*, oxidative stress, L-amino acid oxidase, antibacterial, phytophatogen

## Abstract

*Ralstonia solanaceraum* is the quarantine plant pathogenic bacterium that causes bacterial wilt in over 200 host plants, which include economically important crops such as potato, tomato, tobacco, banana, and ginger. Alternative biological methods of disease control that can be used in integrated pest management are extensively studied. In search of new proteins with antibacterial activity against *R. solanacearum*, we identified L-amino acid oxidases (LAOs) from fruiting bodies of *Amanita phalloides* (*Ap*LAO) and *Infundibulicybe geotropa* (*Cg*LAO). We describe an optimized isolation procedure for their biochemical characterization, and show that they are dimeric proteins with estimated monomer molecular masses of 72 and 66 kDa, respectively, with isoelectric point of pH 6.5. They have broad substrate specificities for hydrophobic and charged amino acids, with highest K_m_ for L-Leu, and broad pH optima at pH 5 and pH 6, respectively. An enzyme with similar properties is also characterized from the mycelia of *I. geotropa* (*Cg*mycLAO). Fractionated aqueous extracts of 15 species of mushrooms show that LAO activity against L-Leu correlates with antibacterial activity. We confirm that the LAO activities mediate the antibacterial actions of *Ap*LAO, *Cg*LAO, and *Cg*mycLAO. Their antibacterial activities are greater against Gram-negative *versus* Gram-positive bacteria, with inhibition of growth rate, prolongation of lag-phase, and decreased endpoint biomass. In Gram-positive bacteria, they mainly prolong the lag phase. These *in vitro* antibacterial activities of *Cg*LAO and *Cg*mycLAO are confirmed *in vivo* in tomato plants, while *Ap*LAO has no effect on disease progression *in planta*. Transmission electron microscopy shows morphological changes of *R. solanacearum* upon LAO treatments. Finally, broad specificity of the antibacterial activities of these purified LAOs were seen for *in vitro* screening against 14 phytopathogenic bacteria. Therefore, these fungal LAOs show great potential as new biological phytoprotective agents and show the fruiting bodies of higher fungi to be a valuable source of antimicrobials with unique features.

## Introduction

The search for new antibacterial agents is especially important against plant pathogenic bacteria where there are no effective chemical or biological agents available for plant protection (Payne et al., 2007; Sahu et al., 2017). One such plant pathogen is the quarantine bacterium *Ralstonia solanacearum* (Smith, 1896) Yabuuchi et al., 1996, which is the active agent for bacterial wilt in the plant family *Solanaceae*. *R. solanacearum* is a species complex that can infect over 200 host plants, which include economically important crops such as potato, tomato, eggplant, tobacco, banana, pelargonium, and ginger (Allen et al., 2005). Overall, *R. solanacearum* results in approximately US$ 950 million annual losses worldwide. The most affected countries are China, Bangladesh, Uganda and Bolivia, which suffer between 30 and 90% annual crop losses, which can rise to 98% during crop storage (Allen et al., 2005; Yuliar et al., 2015).

L-amino acid oxidases (LAOs; E.C. 1.4.3.2) are enzymes that catalyze the oxidative deamination of L-amino acids to their corresponding α-keto acids, with the generation of ammonia and hydrogen peroxide. They are flavoenzymes, and they show high stereospecificity toward L-isomers of amino acids (Lukasheva et al., 2011; Hossain et al., 2014). LAOs are widely distributed in nature, and they fulfill a wide spectrum of biological roles in nitrogen metabolism and in the protection against antagonists, with antimicrobial activities representing one of their main functions. Moreover, LAOs represent a major component of snake venoms, where they serve as toxins, which have been studied in great detail to date. These are flavin adenine dinucleotide or flavin mononucleotide binding proteins, with molecular masses from 50 to 300 kDa, and isoelectric points between pH 4.0 and 9.4. They are usually glycosylated and form non-covalently associated homodimers. Most LAOs have a broad range of substrate specificities, with preference for hydrophobic amino-acid substrates, including L-Phe, L-Leu, L-Trp, L-Met, and L-Ile. On the other hand, some LAOs have very narrow substrate specificities, with high preference for basic L-amino acids, such as L-Lys oxidase from *Trichoderma viride*. The biological effects of LAOs are mediated through their enzymatic activity in two ways: (i) via elimination of amino acids from the extracellular environment, which can cause nutrient deficiency, and/or (ii) via binding to the surface of cells and generating high local concentrations of hydrogen peroxide, which can lead to cell death (Du and Clemetson, 2002; Lukasheva et al., 2011; Guo et al., 2012; Hossain et al., 2014). Several snake venom LAOs have strong antibacterial activities that show wide variations in their selectivities and specificities against Gram-positive and/or Gram-negative bacteria (Guo et al., 2012; Izidoro et al., 2014). Only a few LAOs have been isolated from fungi, however, a recent screening of LAO activities in fungal fruiting bodies revealed that they represent a new rich and readily accessible source of versatile and robust enzymes with LAO activities (Žun et al., 2017).

Although there have been extensive studies on biological control of *R. solanaceraum* (Anith et al., 2004; Ji et al., 2005; Messiha et al., 2007; Hong et al., 2011; Maji and Chakrabartty, 2014; Yuliar et al., 2015), to date there are no efficient chemical or biological agents available for its control. So far, only a few fungal proteins have been tested in the field of agricultural crop protection, including tamavidin (Takakura et al., 2012), mycocypins (Šmid et al., 2013, 2015) and different lectins (Pohleven et al., 2011; Sabotič et al., 2016). The only example of mushroom proteins that are active against bacterial plant pathogens was reported by Zheng et al. (2010), who isolated an antibacterial protein from dried fruiting bodies of the mushroom *Clitocybe sinopica*, however, they did not perform any *in vivo* tests (Zheng et al., 2010). A screening study for antibacterial activities against *R. solanacearum* that included 150 aqueous extracts of fungal fruiting bodies from 94 different species revealed complete growth inhibition of *R. solanacearum in vitro* for 11 of these extracts. Two extracts were selected for isolation and characterization of the antibacterial active substance. One was from the poisonous death cap *Amanita phalloides* (Fries) Link (1833), which showed broad antibacterial activity *in vitro* against Gram-negative bacteria but no *in vivo* activity. The other was from the edible trooping funnel mushroom *Infundibulicybe geotropa* (Buillard ex DeCandolle) Harmaja (2003), which showed antibacterial activity against different strains of *Ralstonia* spp., and also antibacterial activity *in vivo* for both tomato and potato (Erjavec et al., 2016).

Here, we describe the biochemical characterization of proteins with antibacterial activity against *R. solanacearum* that are isolated from fruiting bodies of *A. phalloides* and *I. geotropa*, and from *I. geotropa* cultured mycelia. *I. geotropa* has been reclassified taxonomically from *Clitocybe geotropa*, however, the abbreviation of the protein name (*Cg*LAO) as well as that from the mycelium (*Cg*mycLAO) is maintained here for continuity of the characterization of these proteins, as their apoptosis-inducing activities on cancer cell lines were published previously (Pišlar et al., 2016). We determined the antibacterial activities of these isolated proteins against *R. solanacearum* both *in vitro* and *in vivo*. We finally indicate a possible mechanism of action for the *I. geotropa* protein through electron microscopy analysis of *R. solanacearum* cells in the presence of the purified protein fraction from *I. geotropa*, and through determining the effects of these isolated antibacterial proteins on model Gram-positive and Gram-negative bacteria.

## Materials and Methods

### Materials and Fungal Samples

The L-amino acids L-Thr, L-Gly, L-Ala, L-Met, L-Phe, and L-Tyr were from Serva (Heidelberg, Germany), L-Arg, L-Val, and L-Trp from Fluka (Buchs, Switzerland), L-Pro from Merck (Darmstadt, Germany) and L-His, L-Lys, L-Asp, L-Glu, L-Ser, L-Asn, L-Gln, L-Cys, L-Ile, and L-Leu from Sigma-Aldrich (St. Louis, MO, United States). Complete supplement mixture (CSM) was from Formedium (Norfolk, United Kingdom), and peptide N-glycosidase F was from Roche Diagnostics (Basel, Switzerland). Horseradish peroxidase, catalase and other reagents (all of analytical or sequencing grade) were from Sigma-Aldrich (St. Louis, MO, United States). Glutaraldehyde, osmium tetroxide, and uranyl acetate were from SPI Supplies (West Chester, PA, United States), Paraformaldehyde, epoxy resins agar 100 and lead citrate were from Agar Scientific (Essex, United Kingdom). Yeast extract and casamino acids were from Difco (Detroit, MI, United States), proteose peptone, bacto-peptone and agar from Oxoid (Basingstoke, United Kingdom), glucose and sucrose from Kemika (Zagreb, Croatia), malt extract from BioMerieux (Marcy l’Etoile, France), and M17 from Merck (Darmstadt, Germany). Fruiting bodies of *Agaricus bisporus* (J. E. Lange) Imbach 1946, *Macrolepiota procera* (Scop.) Singer 1948, *Coprinopsis cinerea* (Schaeff.) Redhead, Vilgalys and Moncalvo 2001, *Amanita phalloides* (Vaill. ex Fr.) Link 1833, *Amanita rubescens* Pers. 1797, *Hygrophorus erubescens* (Fr.) Fr. (1838), *Hygrophorus russula* (Schaeff. ex Fr.) Kauffman (1918), *Infundibulicybe geotropa* (Bull.) Harmaja (2003), *Clitocybe nebularis* (Batsch) P. Kumm. (1871), *Lepista nuda* (Bull.) Cooke (1871), *Tricholoma saponaceum* (Fr.) P. Kumm. (1871), *Imleria badia* (Fr.) Vizzini (2014), *Suillus variegatus* (Sw.) Richon and Roze (1888), *Cantharellus cibarius* Fr. (1821), and *Tuber mesentericum* Vittad. (1831) (Table 1) were collected in their natural habitat in forest stands or grasslands in central and western Slovenia and frozen at −20°C. The taxonomic classification follows the Index Fungorum database^1^.

**TABLE 1:** Antibacterial activities against *R. solanacearum* and LAO activities (at pH 5.5) in the fungal fruiting body extracts and their gel filtration fractions.

Family	Species	Estimated Mw of active fraction (kDa)	*In vitro* antibacterial activity		LAO activity of gel filtration fractions		
			Extract	Active	In-gel	In-solution	
				fraction	vs. amino acids in CSM	vs. amino acids in CSM	vs. L-Leu
Agaricaceae	*Agaricus bisporus*	25	–	–	+	+	–
	*Macrolepiota procera*	na	–	–	–	–	–
Psathyrellaceae	*Coprinopsis cinerea*	na	–	–	–	–	–
Amanitaceae	*Amanita phalloides*	120	+	+	+	+	+
	*Amanita rubescens*	na	nd	–	–	–	–
Hygrophoraceae	*Hygrophorus erubescens*	na	–	–	–	–	–
	*Hygrophorus russula*	na	–	–	–	–	–
Tricholomataceae	*Infundibulicybe geotropa*	80	+	+	+	+	+
	*Clitocybe nebularis*	30	+	±	+	+	–
	*Lepista nuda*	150	–	+	+	–	–
	*Tricholoma saponaceum*	na	+	–	–	–	–
Boletaceae	*Imleria badia*	30	nd	–	+	+	–
Suillaceae	*Suillus variegatus*	na	–	–	–	–	–
Hydnaceae	*Cantharellus cibarius*	na	nd	–	–	–	–
Tuberaceae	*Tuber mesentericum*	50	–	+	+	+	+

*Details of the elution profiles for the LAO and antibacterial activities of the gel filtration fractions are illustrated in Supplementary Figure S4. ±, Antibacterial activity detected in a different fraction than LAO activity. na, not applicable (no activity); nd, not determined. CSM, complete supplement mixture.*

### Isolation of *I. geotropa* LAO From Fruiting Bodies and Vegetative Mycelia

After thawing, 240 mL of crude aqueous extract was pressed out of 500 g of the fruiting bodies. After addition of 2 M NaSCN and 3 M urea, the extract was concentrated by ultrafiltration using 3-kDa cut-off membranes. The precipitated material was removed by centrifugation at 8000 × *g* for 20 min at 4°C. The samples were then divided into three equal portions, with each applied to a preparative gel filtration chromatography column (4 × 110 cm; flow rate, 42 mL/h; fraction volume, 17 mL) using Sephacryl S200 (GE Healthcare Life Sciences, Uppsala, Sweden) equilibrated in 0.02 M Tris–HCl, pH 7.5, with 0.3 M NaCl and 3 M urea (buffer A). The fractions with antibacterial activity were pooled, concentrated by ultrafiltration, and dialyzed against 50 mM phosphate buffer, pH 6.8 (buffer B), which contained 0.85 M ammonium sulfate. They were then applied to a hydrophobic interaction chromatography column (3 × 35 cm; flow rate, 19.2 mL/h; fraction volume, 12 mL) using phenyl-Sepharose (GE Healthcare Life Sciences, Uppsala, Sweden) equilibrated in buffer B. The bound protein was eluted using a linear gradient of ammonium sulfate, from 0.85 to 0 M in buffer B (1200 mL), followed by a gradient of 0 to 20% ethanol in buffer B (400 mL). The fractions with the highest antibacterial activities were pooled and concentrated by ultrafiltration. This is an optimized protocol from the previously published *Cg*LAO purification (Pišlar et al., 2016) with highly improved yield.

*Infundibulicybe geotropa* mycelia were cultivated as described previously (Erjavec et al., 2016), collected by filtration through cheesecloth, and centrifuged (8000 × *g* for 10 min at 4°C) and stored at −20°C until use. The solid mycelia (15 g) were homogenized in liquid nitrogen, and the protein from this hyphal powder was extracted overnight in 100 mL buffer A. The insoluble material was removed by centrifugation (16000 × *g* for 5 min at 4°C), and the resulting crude *I. geotropa* mycelium extract was subjected to the same purification procedure as the crude fruiting body extracts.

### Isolation of *A. phalloides* LAO From Fruiting Bodies

The isolation procedure for *A. phalloides* LAO (*Ap*LAO) was the same as that used for *I. geotropa* LAO (*Cg*LAO), except that 2 M NaSCN was omitted in the extract preparation. The two separation steps using gel filtration and hydrophobic interaction chromatography were as described above for *Cg*LAO.

### SDS-PAGE, Two-Dimensional SDS-PAGE, Native PAGE, and Isoelectric Focusing

The proteins were routinely analyzed on 10% polyacrylamide gels under denaturing and reducing conditions, and visualized using Coomassie brilliant blue staining or silver staining. Non-denaturing and non-reducing conditions were used for analyses of protein complexes and LAO activities. Low molecular weight markers of 14.4 to 97 kDa (GE Healthcare, Chicago, IL, United States) were used for molecular mass estimations.

For the two-dimensional SDS-PAGE analysis, the protein was precipitated by trichloroacetic acid/acetone, vacuum dried, and reconstituted in 125 μL rehydration buffer (7 M urea, 2 M thiourea, 30 mM Tris, 0.25% amidosulphobetaine-14, 2.5% 3-[(3-cholamidopropyl)dimethylammonio]-1-propanesulfonate (CHAPS), 0.002% bromophenol blue, 1% ampholytes, 12 μL/mL destreak reagent). A Ettan IPGphor II isoelectric focusing (IEF) system (GE Healthcare, Chicago, IL, United States) was used, and 7 cm Immobiline DryStrip pH gradient (IPG) strips 3-11 NL (GE Healthcare, Chicago, IL, United States) were passively rehydrated overnight with the protein samples. The IEF was performed at 20°C using the following sequential steps: 300 V for 45 min; 300 V to 1000 V linear gradient for 30 min; 1000 V to 5000 V linear gradient for 72 min; and 5000 V to the final 6000 Vh. The current was restricted to 50 μA/strip. The focused IPG strips were exposed to 65 mM dithiothreitol followed by 135 mM iodoacetamide in 75 mM Tris–HCl buffer with 6 M urea, 4% sodium dodecyl sulfate, 30% glycerol and 0.002% bromophenol blue. Then, 10% polyacrylamide gels were used for the second dimension of the SDS-PAGE, which were stained by the highly sensitive imidazole-zinc negative staining, as described previously (Fernandez-Patron et al., 1992). The protein spots were excised and stored at −20°C before further processing.

The proteins were analyzed under non-denaturing conditions using blue native PAGE with a Novex NativePAGE Bis-Tris gel system with 4 to 16% gradient protein gels (Life Technologies, Carlsbad, CA, United States), according to the manufacturer instructions. NativeMark unstained protein standards (ThermoFisher Scientific, Waltham MA, United States) was used for the molecular mass estimations.

Isoelectric focusing was carried out with a Pharmacia PhastSystem, using commercial precast pH 3-9 gradient gels (GE Healthcare, Chicago, IL, United States) following the manufacturer instructions. Alternatively, precast Novex pH 3-7 IEF protein gels (Life Technologies, Carlsbad, CA, United States) were used. Marker proteins with pI values from 3.5 to 9.3 were used for calibration (GE Healthcare, Chicago, IL, United States).

### Protein Glycosylation Analysis

The glycosylation of proteins was assessed using treatment with peptide N-glycosidase (Magnelli et al., 2011). Protein samples were denatured by heating to 100°C for 10 min in 1% sodium dodecyl sulfate, and were then mixed with 50 mM Na_2_HPO_4_, pH 7.5, with 1.5% CHAPS before peptide N-glycosidase F (3 U) was added to half of each sample; the other half of each sample served as the controls. Both of these samples were incubated at 37°C for 24 h, and then subjected to SDS-PAGE analysis under reducing conditions.

### Mass Spectrometry and N-Terminal Sequence Analysis

The proteins were resolved in one- or two-dimensional SDS-PAGE, and the individual bands and spots were excised. After in-gel trypsin digestion, they were identified by peptide mass fingerprinting (Ganten et al., 2006) using an ion trap mass spectrometer (1200 series HPLC-Chip-LC/MSD Trap XCT Ultra; Agilent Technologies, Santa Clara, CA, United States). Database searches were performed using the Mascot in-house server for the MS/MS ion searches.

The N-terminal amino-acid sequences of the proteins were determined by automated amino-acid sequencing using Procise Protein Sequencing System 492 (Applied Biosystems, Foster City, CA, United States). The proteins were resolved by SDS-PAGE, electroblotted onto polyvinylidene difluoride membranes, and stained with Coomassie blue. The individual protein bands were then excised and analyzed (Reim and Speicher, 2001).

### LAO Activity Assay

The LAO activities of the protein samples were determined spectrophotometrically, as described previously (Kishimoto and Takahashi, 2001). Briefly, each protein sample was mixed with the reaction mixture that contained the substrate (5 mM L-amino acid or amino acids in 0.1% CSM), 2 mM o-phenylenediamine and 0.81 U/mL horseradish peroxidase in 0.1 M bis-Tris, pH 5.5, in 96-well microplates. Absorbance at 420 nm was measured at constant time intervals over 30 min at 30°C in a microplate reader (Infinite M1000; Tecan, Grödig, Austria). For substrate specificity analysis, the individual L-amino acids (5 mM) were used in the reaction mixture. For inhibition of LAO activity, ascorbic acid was added to the reaction mixture to final concentrations of 0.1 to 5 mg/mL.

For the optimum pH analysis for the LAO activities, these were analyzed as described above with L-Leu as the substrate and in phosphate-citrate buffer from pH 2.6 to pH 7.8, K-phosphate buffer from pH 6 to pH 9, and carbonate-bicarbonate buffer from pH 9 to pH 11.

In-gel analysis of the LAO activities was performed as described previously (Žun et al., 2017). Briefly, non-denatured samples were subjected to SDS-PAGE in 10% polyacrylamide gels. After electrophoresis, the gels were washed in 0.1 M bis-Tris, pH 5.5, and then incubated in the reaction mixture containing substrate (5 mM L-amino acid or amino acids in 0.1% CSM), 1 mM o-phenylenediamine and 0.5 U/mL horseradish peroxidase, in the same buffer at room temperature in the dark for 1 to 20 h. After stopping the reaction by adding 2 M H_2_SO_4_, the brown bands of the LAO activity were analyzed using an image scanner (Canon LiDE 110, Middlesex, United Kingdom).

### Bacterial Cultures and Inoculum Preparation

The National Collection of Plant Pathogenic Bacteria strain 4156 *Ralstonia solanacearum* (Smith, 1896) Yabuuchi et al., 1996 (phylotype IIB, race 3, biovar 2) (Wullings et al., 1998) was isolated from potatoes in 2001 in Netherlands. This was used as the study reference isolate for the *in vitro* testing of antibacterial activity. *R. solanacearum* were grown at 28°C on yeast peptone glucose (YPG) agar plates (per liter: 5 g yeast extract, 5 g proteose peptone, 10 g glucose, 12 g agar; pH 7.2-7.4). Bacterial suspensions were prepared in 0.01 M phosphate-buffered saline (PBS) (per liter: 1.071 g Na_2_HPO_4_, 0.4 g NaH_2_PO_4_7H_2_O, 8.0 g NaCl; pH 7.2). The bacterial concentrations were determined according to absorbance at 595 nm (A_595_), and they were dilution plated on YPG agar (CFU/mL). Alternatively, BG medium (per liter: 10 g bacto-peptone, 1 g yeast extract, 1 g casamino acids, 5 g glucose) was used for *R. solanacearum* cultivation.

### *In vitro* Testing of Antibacterial Activity Against *R. solanacearum* and Other Plant Pathogenic Bacteria

The *in vitro* testing for antibacterial activities was performed in microtiter plates following a previously published protocol (Erjavec et al., 2016). Briefly, the 200 μL testing wells contained YPG (75 μL), the suspension of *R. solanacearum* (10^7^ cells/mL, 75 μL), 0.01 M PBS (42.5 μL) and the mushroom extract or protein fraction (7.5 μL). The following controls were included for each plate: positive control (no extract added) indicating the normal growth of *R. solanacearum* in these conditions; negative control (no extract and *R. solanacearum* added); and controls of extract sterility (no *R. solanacearum* added). All of the controls were supplemented to 200 μL with 0.01 M PBS. Each sample (i.e., mushroom extract) and the controls were tested in at least two parallel wells. After 24 h, absorbance at 595 nm was measured and 30 μL of the mixture from each well was plated onto fresh YPG agar plates, to determine whether the effects were bactericidal (i.e., no bacterial growth observed when transferred onto fresh medium) or bacteristatic (i.e., bacterial growth observed when transferred onto fresh medium). The effects of the fungal extracts on selected plant pathogenic bacteria [*Dickeya* spp. (NIB B16 and NIB S1), *Dickeya fangzhongdai* (DSMS 101947), *Erwinia amylovora* (NCPPB 683), *Pseudomonas syringae* pv. *syringae* (NCPPB 281), *Clavibacter michiganensis* subsp *michiganensis* (NCPPB 2979), *Agrobacterium tumefaciens* (NCPPB 2437), *Enterobacter* sp. (NCCPB 4168), *Pectobacterium atrosepticum* (NIB Z 620), *Pectobacterium carotovorum* (NIB Z 623), *Dickeya chrysanthemi* (NCPPB 402), *Escherichia coli* (GSPB 48), *Ralstonia mannitolilytica* (CFBP 6737), and *Xanthomonas arboricola* pv. pruni (NCPPB 416)] were tested in media that support good bacterial growth, which included casamino acid-peptone-glucose (CPG) medium (per liter: 1 g casamino acids, 10 g peptone, 5 g glucose), King’s medium B (KB; per liter: 20 g proteose peptone, 1.5 g K_2_HPO_4_, 1.5 g MgSO4 7H_2_O, 10 mL glycerol), nutrient broth yeast extract (NBYE; per liter: 8 g nutrient broth, 5 g yeast extract, pH 7.5) and YPG (Schaad et al., 2001). *Ap*LAO, *Cg*LAO and the LAO extracted from mycelia of *I. geotropa* (*Cg*mycLAO) were tested for their effect on the growth of *R. solanacearum* using the highest concentration available for each sample at different concentrations, as indicated in Table 3.

The combined effects of the purified LAO enzymes and catalase (1000 U/mL) on *R. solanacearum* were tested in liquid YPG and BG media.

### *In vitro* Testing of Antibacterial Activities Against *Escherichia coli* and *Lactococcus lactis*

The Gram-negative bacterium *Escherichia coli* DH5α and the Gram-positive bacterium *Lactococcus lactis* NZ9000 were used to define the antimicrobial mechanism of the LAO activities. Growth curves were followed in rich media, S.O.C. (2% tryptone, 0.5% yeast extract, 10 mM NaCl, 2.5 mM KCl, 10 mM MgSO_4_, 0.4% glucose) for *E. coli*, and GM17 (M17 from Merck supplemented with 0.5% glucose) for *L. lactis*. Overnight cultures were diluted 100-fold in the corresponding fresh medium, to which the filter sterilized LAO samples were added at different concentrations (*Ap*LAO, 64-0.08 μg/mL; *Cg*LAO, 99-1.6 μg/mL; *Cg*mycLAO, 129-21 μg/mL). Alternatively, *E. coli* growth was followed in M9 minimal medium (0.24 M Na_2_HPO_4_, 0.24 M KH_2_PO_4_, 0.09 M NaCl, 0.19 M NH_4_Cl, 1 mM MgSO_4_, 0.1 mM CaCl_2_, 2% glucose) without or with 5 mM L-Leu. The effects of catalase addition were determined in the rich medium using bovine catalase at 1000 U/mL. Growth curves (as triplicates) were followed at 30°C by measuring A_595_ in 96-well plates using a microplate reader (Sunrise; Tecan, Grödig, Austria) and the XFluor4 software, and analyzed using DMFit Microsoft Excel Add-In, version 3.5 (Baranyi and Roberts, 1994).

### Tomato Pathogenicity Test

Tomato pathogenicity tests were used to determine the *in vivo* activities of the *A. phalloides* and *I. geotropa* extracts and the purified LAO enzymes. The same protocols were used as described by Erjavec et al. (2016). Briefly, tomato plants (*Solanum lycopersicon* cv. “Moneymaker”) were used as the test plant. The plants were inoculated at the two-to-three true-leaf stage with mixtures of the *R. solanacearum* suspensions and mushroom extracts or protein samples (10:1 ratio). The bacterial concentration in the inoculation suspension was 10^5^ CFU/mL, and the concentrations of the purified LAO enzymes were 2.14 mg/mL for *Ap*LAO, 3.3 mg/mL for *Cg*LAO, and 4.3 mg/mL for *Cg*mycLAO. The *R. solanacearum* suspension and 0.01 M PBS were used for the inoculation of the positive and negative control plants, respectively. Using a sterile needle (Icogamma plus, 0.6 mm × 25 mm; Novico, Italy), each suspension was inoculated between the cotyledons, with approximately 20 μL of each suspension used per plant.

In total, 42 plants were inoculated with each extract, 42 plants with the positive controls, and 20 plants with the negative controls. After the inoculations, the plants were grown at a 28°C day temperature, with a 16 h photoperiod at 90 μmol m^–2^ s^–1^ photon irradiance, and at a 20°C night temperature. The severities of the symptoms were evaluated regularly over 14 days, following the numerical grades of Winstead and Kelman (1952): 0 (no symptoms), 1 (one leaf wilted), 2 (2-3 leaves wilted), 3 (all leaves except the tip of the plant wilted), 4 (all leaves and the tip of the plant wilted), and 5 (plant dead).

Chi-squared tests were used for symptom severities and disease progression in plants inoculated with mixtures of *R. solanacearum* and protein samples, compared with the positive control plants. The area under the disease progress curve (AUDPC) was used as a measure of quantitative disease resistance, as calculated for the pathogenicity tests (Madden et al., 2007) using the R-statistical (R Development Core Team, 2008) Agricolae package (De mendiburu, 2020).

### Transmission Electron Microscopy

The ultrastructure of the bacterial cells was examined using transmission electron microscopy. Overnight cultures were mixed with *Cg*LAO (50 μg/mL) for 2 h and compared with untreated cells. The cells were fixed in 3% glutaraldehyde and 1% paraformaldehyde in 0.1 M phosphate buffer, post-fixed in 2% osmium tetroxide, and embedded in epoxy resin. Ultrathin sections were cut and examined under transmission electron microscopy (CM 100; Philips, Amsterdam, Netherlands), operating at 80 kV. Micrographs were recorded with a CCD camera (Orius SC 200; Gatan Inc., United States).

## Results

### Isolation of Antibacterial Substances From Crude Protein Extracts of *Amanita phalloides* and *Infundibulicybe geotropa*

**FIGURE 1:**
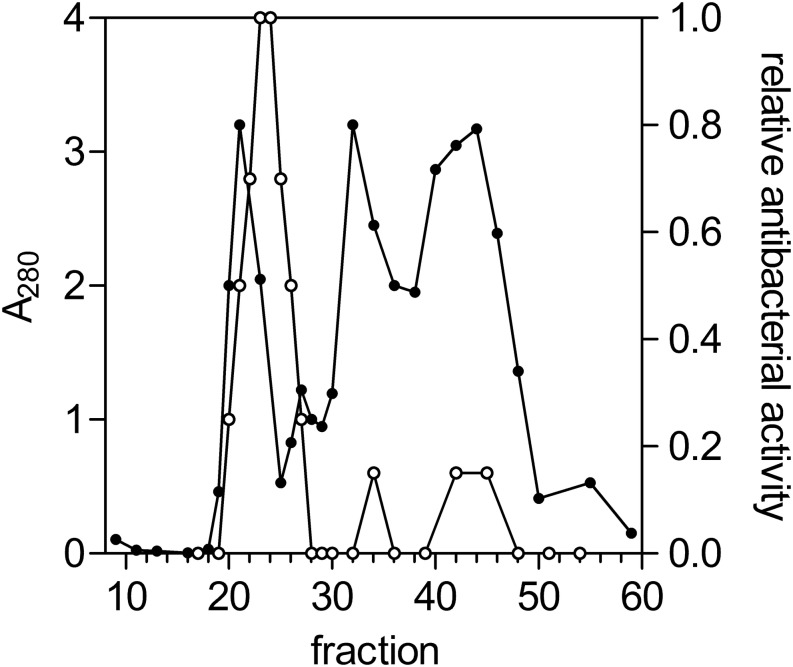
Gel-filtration chromatography of the *Infundibulicybe geotropa* extract. Protein elution profile (A_280_; closed circles) from gel filtration chromatography using Sephacryl S200, for relative antibacterial activity against *Ralstonia solanacearum* (open circles). Fractions 21 to 25 were collected and further purified.

**FIGURE 2:**
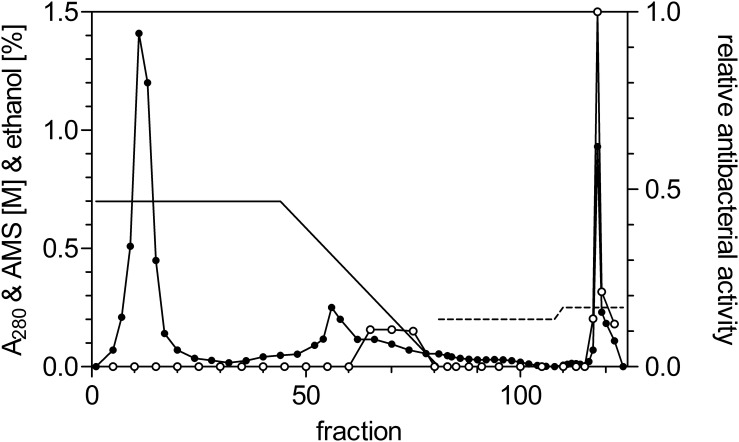
Hydrophobic-interaction chromatography of the fractions with antibacterial activity against *R. solanacearum* from the gel filtration of the *I. geotropa* extract. Protein elution profile (A_280_; closed circles) from hydrophobic interaction chromatography using Phenyl-Sepharose for relative antibacterial activity against *R. solanacearum* (open circles). Solid line, ammonium sulfate gradient; dotted line, ethanol concentration. Fractions 118–120 were collected and concentrated by ultrafiltration.

**FIGURE 3:**
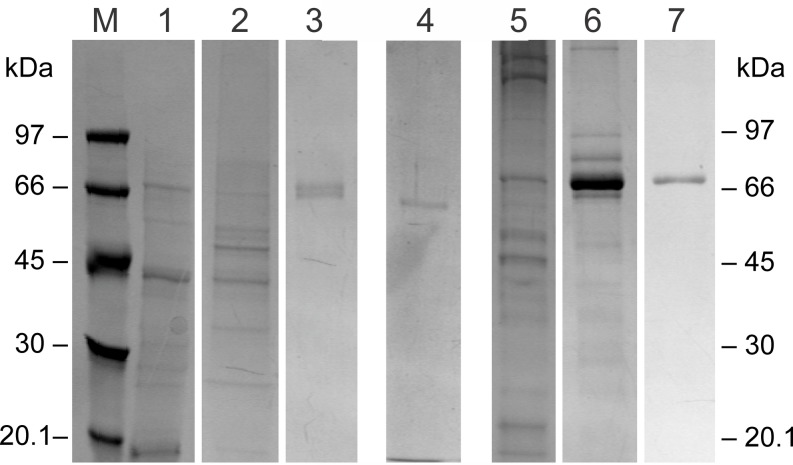
Representative SDS-PAGE analysis of the fractions showing antibacterial activity after the two-step purification procedure of the *I. geotropa* and *A. phalloides* extracts. SDS-PAGE in a 10% polyacrylamide gel under denaturing conditions visualized by Coomassie blue staining. Lane M, molecular mass markers; lane 1, crude aqueous extract of *I. geotropa* fruiting bodies; lane 2, gel filtration fractions 21–25 of *Cg*LAO; lane 3, fraction 118 after hydrophobic-interaction chromatography of *Cg*LAO; lane 4, purified *Cg*mycLAO; lane 5, crude aqueous extract of *A. phalloides* fruiting bodies; lane 6, gel filtration fractions 23–32 of *Ap*LAO; lane 7, fractions 113–117 after hydrophobic-interaction chromatography of *Ap*LAO.

Proteins that showed antibacterial activities against *R. solanacearum* were isolated from the fruiting bodies of *A. phalloides* and *I. geotropa* using a two-step procedure of gel filtration (Figure 1) and hydrophobic-interaction chromatography (Figure 2). These yielded purified proteins of 72 and 66 kDa from A. *phalloides* and *I. geotropa*, respectively, as shown by SDS-PAGE analysis (Figure 3). For the preparation of the crude protein extract from *A. phalloides*, little benefit was seen for the inclusion of NaSCN with the 3 M urea, and so this potential activation step of NaSCN was omitted from the protocol. On the other hand, for the preparation of the crude protein extract from *I. geotropa* fruiting bodies, the addition of 2 M NaSCN with the 3 M urea resulted in an approximately 10-fold increase in the total LAO activity of the extract, so this step was included in the purification scheme.

In a typical preparation, 3.8 and 9.0 mg of the antibacterial proteins were obtained from 500 g of *A. phalloides* and *I. geotropa* fruiting bodies, respectively.

### The Proteins With Antibacterial Activity Are L-Amino Acid Oxidases

**FIGURE 4:**
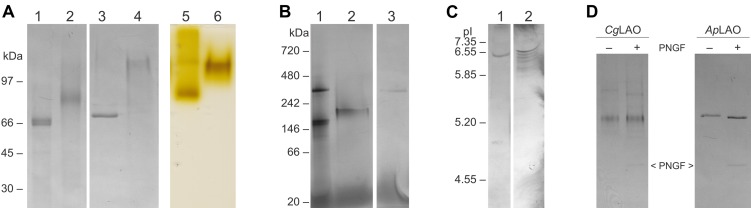
Representative polyacrylamide electrophoretic analyses of purified proteins with antibacterial activity from *I. geotropa* and *A. phalloides*. **(A)** SDS-PAGE analysis in a 10% polyacrylamide gel under denaturing and non-denaturing conditions showing Coomassie blue staining (lanes 1–4) and H_2_O_2_ production (LAO activity; lanes 5, 6). Lanes 1, 2, 5, *Cg*LAO; lanes 3, 4, 6, *Ap*LAO; lanes 1, 3, samples heated to 100°C for 10 min; lanes 2, 4, samples incubated at room temperature. Lanes 5, 6, LAO activities for *Cg*LAO (lane 5) and *Ap*LAO (lane 6). For H_2_O_2_ production, following SDS-PAGE under non-denaturing conditions in a 10% polyacrylamide gel, the LAO activity was revealed by staining for H_2_O_2_ production for 1 h using the *o*-phenylenediamine–horseradish peroxidase system with 5 mM L-Leu as substrate in 0.1 M bis-Tris, pH 5.5. **(B)** Blue native PAGE analysis. Lane 1, purified *Cg*LAO; lane 2, purified *Ap*LAO; lane 3, purified *Cg*mycLAO. **(C)** Isoelectric focusing analysis. Lane 1, purified *Cg*LAO; lane 2, purified *Ap*LAO. **(D)** SDS-PAGE analysis for deglycosylation using peptide-N-glycosidase F (PNGF) in a 10% silver-stained polyacrylamide gel. Left, purified *Cg*LAO; right, purified *Ap*LAO.

Under native conditions, the molecular masses of the purified antibacterial proteins from *A. phalloides* and *I. geotropa* were estimated to be in the 120 to 130 kDa range from the elution volumes on a calibrated gel filtration column (data not shown), which suggested that both proteins form dimers. Furthermore, apparent molecular masses in the 180 to 300 kDa range were obtained using native PAGE analysis (Figure 4B). The antibacterial protein purified from *A. phalloides* fruiting bodies ran at 220 kDa as a diffuse band, whereas two bands of approximately 180 and 360 kDa were observed for the antibacterial protein purified from *I. geotropa* fruiting bodies. The bands were excised from native PAGE, with the proteins eluted overnight by diffusion from gel pieces and the antibacterial activities against *Ralstonia solanacearum* were confirmed *in vitro*. The molecular masses revealed by PAGE analysis under native and denaturing conditions (Figure 4), together with the gel filtration of the crude extracts and the purified proteins, indicated that the antibacterial proteins from both of these species form higher molecular aggregates or multimers in the fruiting body extracts. Furthermore, during the purification procedures these higher molecular protein complexes dissociated mostly into dimers in solution.

Analysis of the isoelectric points revealed that both of these proteins have similar isoelectric points, at approximately pH 6.5. The antibacterial protein from *A. phalloides* showed higher heterogeneity (Figure 4C). Several bands were observed for the *A. phalloides* protein with isoelectric focusing, which were probably the result of glycosylation variants, as N-glycosylation was confirmed for the antibacterial protein from *A. phalloides* (Figure 4D). On the other hand, N-glycosylation was not confirmed for the *I. geotropa* antibacterial protein (Figure 4D).

N-terminal sequencing of both of these proteins did not provide conclusive data to enable primer design for gene sequence retrieval. Therefore, the individual spots from the *I. geotropa* 2D-PAGE separation (Supplementary Figure S1) were cut out, eluted and subjected to mass spectrometry analysis. However, only similarity to a bacterial dihydrolipoyl dehydrogenase (EC 1.8.1.4) with limited coverage was detected, which was not considered a significant hit. On the other hand, using mass spectrometry analysis the protein from *A. phalloides* with antibacterial activity was identified as toxophallin, an LAO that was isolated from *A. phalloides* fruiting bodies (Stasyk et al., 2010). Poor outcome of the N-terminal amino acid sequencing and mass spectrometry peptide fingerprinting is probably the consequence of the fact that genomes of these two fungal species are not yet available and the similarity to characterized LAOs from other organisms is too low to be detected.

L-amino acid oxidase activities were confirmed in the fractions with antibacterial activity after the gel filtration and hydrophobic interaction chromatography for both species. The proteins with antibacterial activities are therefore termed *Ap*LAO and *Cg*LAO. The in-gel LAO activities (Figure 4A, lanes 5, 6) corresponded well to the bands of the non-denatured *Ap*LAO (Figure 4A, lane 4) and *Cg*LAO (Figure 4A, lane 2), as observed under conditions of SDS-PAGE analysis.

### *I. geotropa* Produces LAO in Vegetative Mycelia

A protein with antibacterial activity and LAO activity was also isolated from *I. geotropa* mycelia following the same procedures as for that from the fruiting bodies. Both the mycelium extract and the gel filtration fractions showed LAO activities that completely inhibited *R. solanacearum* growth (Supplementary Figure S2). Furthermore, concentrated spent medium from mycelium growth had no effect on *R. solanacearum* growth indicating that the antibacterial LAO is not secreted. The LAO isolated from the cultured vegetative mycelia of *I. geotropa* was termed *Cg*mycLAO, and it showed very similar characteristics to that from *I. geotropa* fruiting bodies (*Cg*LAO). *Cg*mycLAO showed a single band of approximately 400 kDa on native PAGE (Figure 4B), and one prominent band of approximately 65 kDa on SDS-PAGE (Figure 3). Identification of *Cg*mycLAO by mass spectrometry analysis was also not successful, as no similarity to identified peptides was found in the available databases.

### L-Amino Acid Oxidase Activity

**FIGURE 5:**
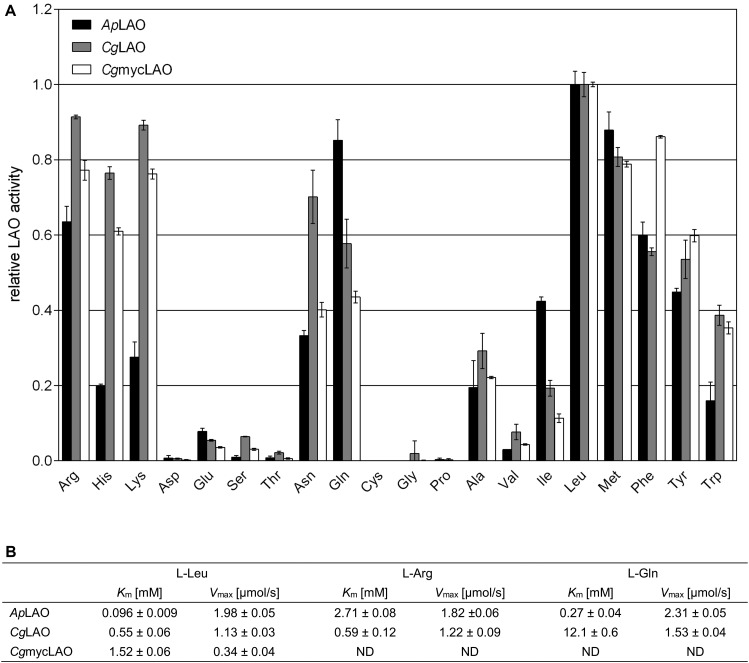
Substrate specificities and kinetic properties of *Ap*LAO, *Cg*LAO and *Cg*mycLAO. **(A)** Quantitative analysis of different L-amino acid substrates (5 mM) at pH 7.5 and 37°C. Data are means ± standard deviation normalized to L-Leu, the optimal substrate for both enzymes. **(B)** K_m_ and V_max_ were determined experimentally and using Michaelis-Menten equation.

The *Ap*LAO, *Cg*LAO, and *Cg*mycLAO showed similar broad substrate specificities for L-amino acids with hydrophobic and charged side chains (Figure 5A). They all showed their highest LAO activity against L-Leu, with *K*_m_ in the low millimolar range (Figure 5B). *Ap*LAO showed approximately 2-fold higher specific activity compared to *Cg*LAO. Addition of the antioxidant agent ascorbic acid (at 0.1 mg/mL or higher) inhibited *Ap*LAO, *Cg*LAO, and *Cg*mycLAO activities *in vitro* (Supplementary Figure S3) similarly, as previously shown for toxophallin (Stasyk et al., 2010).

**FIGURE 6:**
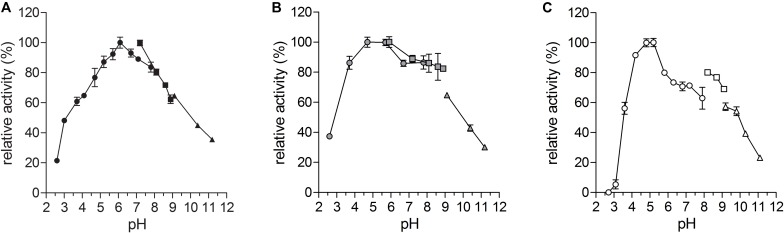
pH optima of *Ap*LAO, *Cg*LAO and *Cg*mycLAO. pH range for activity of *Ap*LAO **(A)**, *Cg*LAO **(B)**, and *Cg*mycLAO **(C)** as analyzed using L-Leu as substrate: pH 2.6 to pH 7.8, phosphate-citrate buffer (circles); pH 6 to pH 9, K-phosphate buffer (squares); pH 9 to pH 11, carbonate-bicarbonate buffer (triangles). Data are means ± standard deviation.

All three enzymes had a broad pH optimum (Figure 6), which peaked at pH 6 for *Ap*LAO and at pH 5 for *Cg*LAO and *Cg*mycLAO. Furthermore, they had a wide pH range for their activities, with >50% enzymatic activity in the pH range from pH 3 to pH 10.

### L-Leu LAO Activity Correlates With Antibacterial Activity in the Fractionated Extracts of Fungal Fruiting Bodies

This study is a follow-up to the screening study of antibacterial activities against *R. solanacearum* in mushrooms (Erjavec et al., 2016), therefore, analysis of LAO mediated antibacterial activity was broadened in order to assess, whether it is widely distributed among fungal species or limited to a few species. The fruiting body extracts of 15 species of mushrooms covering one ascomycete and seven basidiomycete families were fractionated using gel filtration chromatography (Sephacryl S300). Their antibacterial activities against *R. solanacearum* and their LAO activities against L-Leu and the defined mixture of amino acids in CSM were determined for the fractions (Table 1 and Supplementary Figure S4). The LAO activities against the CSM amino acids of the fractions detected using the in-gel activity method were also determined spectrophotometrically, with the exception of the *Lepista nuda* fractions, which only showed in-gel LAO activities. The fractions of *A. phalloides*, *I. geotropa*, and *Tuber mesentericum* that showed LAO activity against the CSM amino acids were also active against L-Leu, and the same fractions also had antibacterial activities. On the other hand, the fractions of the *Agaricus bisporus* and *Xerocomus badius* extracts only showed LAO activities against the CSM amino acids, and they were not active against L-Leu and also did not have antibacterial activities. Similarly, the fractions of the *Clitocybe nebularis* extract that showed LAO activity only against the CSM amino acids were not active against L-Leu, and had no antibacterial activities. However, antibacterial activity was detected in one of the other fractions from the *C. nebularis* extract, which indicated the presence of a non-LAO antibacterial activity. Moreover, LAO activity was detected exclusively by the in-gel detection method using the CSM amino acids in one fraction of the *L. nuda* extract, which also showed antibacterial activity.

The LAO activities that were detected in-gel against the CSM amino acid substrate with apparent molecular masses of 50 kDa and above correlated with the antibacterial activities. On the other hand, the fractions that showed LAO activities with apparent molecular masses of 30 kDa or less did not have antibacterial activities.

A lack of antibacterial activity *in vitro* in the fruiting body extracts does not necessarily signify a lack of antibacterial compounds in these extracts. There were no antibacterial activities detected in the whole extracts from *L. nuda* and *T. mesentericum*, although there were antibacterial activities detected in their gel filtration fractions. Conversely, the *T. saponaceum* extract had antibacterial activity, but this was lost upon fractionation.

### LAO Activity Mediates the Antibacterial Effects on *E. coli* and *L. lactis*

**FIGURE 7:**
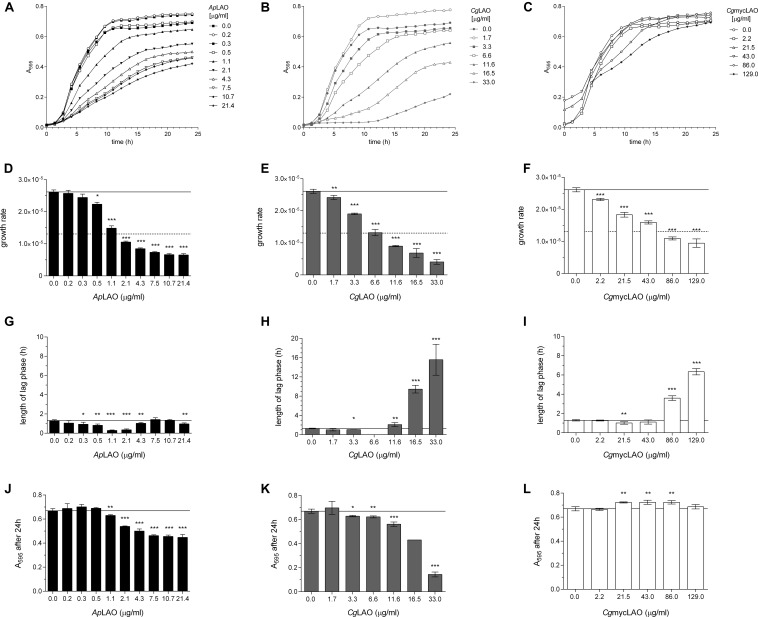
Effects of *Ap*LAO, *Cg*LAO and *Cg*mycLAO on *E. coli* growth measures. Representative *E. coli* growth curves **(A–C)** and derived growth parameters as growth rate **(D–F)**, length of lag phase **(G–I)**, and absorbance after 24 h **(J–L)** in the absence and presence of increasing concentrations of *Ap*LAO **(A,D,G,J)**, *Cg*LAO **(B,E,H,K)** and *Cg*mycLAO **(C,F,I,L)**. Data are means ± standard deviation, with the analysis performed twice, each as three technical repeats. *p < 0.05; **p < 0.01; and ***p < 0.001 (Student’s *t*-tests vs. no addition control). Guiding lines help visualize the control value.

**FIGURE 8:**
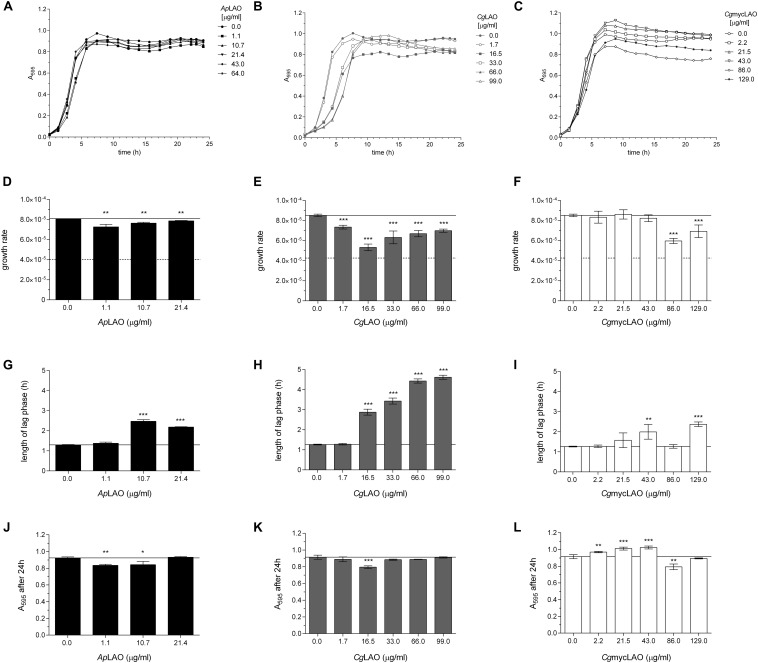
Effects of *Ap*LAO, *Cg*LAO and *Cg*mycLAO on *L. lactis* growth measures. Representative *L. lactis* growth curves **(A–C)** and derived growth parameters as growth rate **(D–F)**, length of lag phase **(G–I)**, and absorbance after 24 h **(J–L)** in the absence and presence of increasing concentrations of *Ap*LAO **(A,D,G,J)**, *Cg*LAO **(B,E,H,K)** and *Cg*mycLAO **(C,F,I,L)**. Data are means ± standard deviation, with the analysis performed twice, each as three technical repeats. *p < 0.05; **p < 0.01; ***p < 0.001 (Student’s *t*-tests vs. no addition control). Guiding lines help visualize the control value.

To determine the mode of action of these enzymes, the effects of *Ap*LAO, *Cg*LAO, and *Cg*mycLAO were examined on the growth of two model bacteria. Their antibacterial activities were greater against the Gram-negative *E. coli* than the Gram-positive *L. lactis* (Figures 7, 8). *Ap*LAO, *Cg*LAO, and *Cg*mycLAO all slowed the growth rates of *E. coli*, and *Cg*LAO and *Cg*mycLAO also significantly prolonged the *E. coli* lag phase, which was up to 10-fold longer. Also, *Ap*LAO and *Cg*LAO, but not *Cg*mycLAO, promoted *E. coli* transition to the stationary phase at a lower optical density (A_595_). On the other hand, the *L. lactis* growth rate and optical density at transition to the stationary phase was less affected by *Ap*LAO, *Cg*LAO and *Cg*mycLAO, and the main effect of all three of these LAOs was for prolongation of the lag phase. The effect was less substantial than for *E. coli*, as the lag phase was at most tripled (by the highest concentration of *Cg*LAO used).

Surprisingly, the catalase-negative *L. lactis* was more resistant to these LAO activities in terms of all of the growth parameters, as compared to *E. coli*, which expresses catalase. Nevertheless, the addition of catalase to the medium alleviated or abolished the effects of these LAO activities on both *E. coli* and *L. lactis* (Supplementary Figure S5). Furthermore, the addition of the preferred substrate of *Ap*LAO, *Cg*LAO, and *Cg*mycLAO to the minimal medium increased the antibacterial activity on *E. coli* of all three of these LAOs, and for all of the growth parameters (Supplementary Figure S6). These results confirm that the antibacterial effects of all three LAOs are indeed a consequence of the LAO activity by oxidative deamination of L-amino acids.

### LAO Activity Mediates the Antibacterial Effects on *R. solanacearum*

**FIGURE 9:**
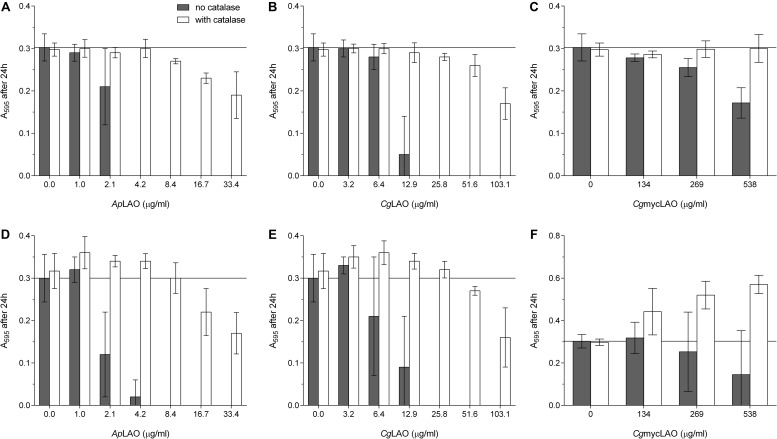
Catalase removes the inhibition of *R. solanacearum* by *Ap*LAO, *Cg*LAO, and *Cg*mycLAO. Representative absorbance after 24 h in the absence and presence of increasing concentrations of *Ap*LAO **(A,D)**, *Cg*LAO **(B,E)** and *Cg*mycLAO **(C,F)** without (closed columns) and with (open columns) addition of catalase. The analyses were carried out in BG medium **(A–C)** and YPG medium **(D–F)**. Data are means ± standard deviation, with the analysis performed twice, each as three technical repeats. Guiding lines help visualize the control value.

The antibacterial activity of *Ap*LAO, *Cg*LAO, and *Cg*mycLAO is due to their enzymatic activity. *Ap*LAO and *Cg*LAO showed similar antibacterial activities against *R. solanacearum* in YPG and BG medium, with the minimum inhibitory concentration of 4.2 μg/mL in BG and 8.4 μg/mL in YPG for *Ap*LAO and 25.8 μg/mL for *Cg*LAO (Figure 9). The inhibitory activities of *Cg*mycLAO were minimal, and were only detected at 20-fold higher concentrations compared to *Cg*LAO. The addition of catalase reduced the inhibitory effects of the LAO activity in a concentration-dependent manner, which confirmed that this inhibitory activity is the consequence of the LAO enzymatic activity (Figure 9).

### Transmission Electron Microscopy of *R. solanacearum*

**FIGURE 10:**
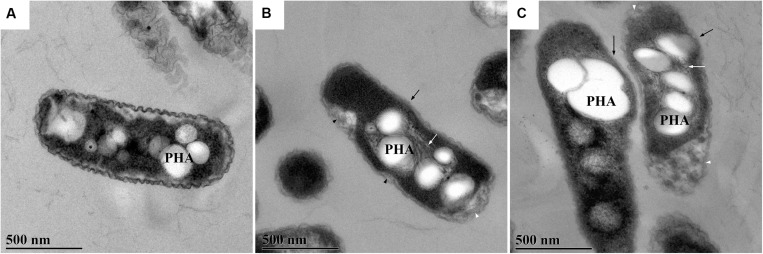
Representative transmission electron microscopy after treatment of *R. solanacearum* with *Cg*LAO. *R. solanacearum* were left untreated **(A)** or were treated **(B,C)** with *Cg*LAO at 50 μg/mL for 2 h. PHA, polyhydroxyalkanoate granules. **(B,C)** White arrows, filaments around granules; black arrows, bulge in the bacterial surface; black arrowheads, detachment of plasma membrane; white arrowheads, large periplasmic spaces at the poles of the cell.

Micrographs of *R. solanacearum* were produced by transmission electron microscopy, and these revealed the influences of *Cg*LAO on the bacterial ultrastructure. The comparisons of the bacterial shapes before (Figure 10A) and after (Figures 10B,C) treatment with *Cg*LAO indicated presence of “bulges” on the cell surface (Figures 10B,C, black arrows) and increased filamentous structure around the PHA granules (Figures 10B,C, white arrows). Control cells had a wrinkled outer membrane with a visible periplasmic space (Figure 10A), which was sometimes enlarged due to invagination of the inner membrane. After the *Cg*LAO treatment, the volume of the periplasmic space appeared to increase mostly at the cell poles (Figures 10B,C, white arrowheads), although the plasma membrane was also seen to be detached from the cell wall in other places (Figure 10B, black arrowheads). The periplasmic space of the *Cg*LAO-treated cells also appeared to contain more granulated material compared to the control cells, and the outer membrane became less wrinkled and looked smooth, and in some cases, amorphous (Figures 10B,C).

### *In vivo* Pathogenicity Tests

**FIGURE 11:**
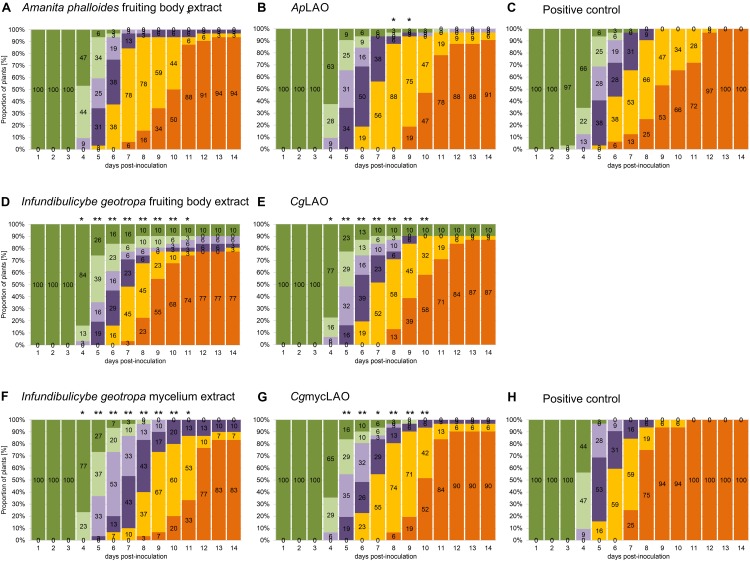
Effects of extracts and purified LAOs on bacterial wilt disease progression in artificially inoculated potted tomato plants of cv. “Moneymaker.” Plants were stem-inoculated with mixture of *R. solanacearum* (10^5^ CFU/mL) and the extracts from the fruiting bodies of *A. phalloides* **(A)** and *I. geotropa* **(D)** and the mycelia of *I. geotropa* **(F)**, or the purified *Ap*LAO **(B)**, *Cg*LAO **(E)** or *Cg*mycLAO **(G)**, or with *R. solanacearum* alone (**C,H**; positive control). The experiments were performed in two growing chambers, one with plants for groups **(A–C)** and the other for groups **(D–H)**. For each time point from 1 to 14 days, the symptoms are expressed as proportion of plants with symptoms for each grade according to Winstead and Kelman (1952): dark green, grade 0 (no symptoms); light green, grade 1 (one leaf wilted); light purple, grade 2 (2–3 leaves wilted); dark purple, grade 3 (all leaves except the tip of the plant wilted); yellow, grade 4 (all leaves and the tip of the plant wilted); orange, grade 5 (plant dead). Within groups, the times at which the distribution of the symptoms differed significantly from the corresponding distribution in the positive control group, are indicated: *p < 0.05 and **p < 0.01 (chi-squared tests). The plants in the negative control groups did not show any symptoms.

For *Cg*LAO and *Cg*mycLAO, the inhibitory activities against *R. solanacearum* disease progression *in vivo* in tomato plants were confirmed, while *Ap*LAO had no such activity *in vivo*. Tomato plants were used in the pathogenicity tests as these are an important *R. solanacearum* host plant, and they are also used as test plants in bacterial diagnostics. The *A. phalloides* and *I. geotropa* extracts and their purified *Ap*LAO, *Cg*LAO, and *Cg*mycLAO fractions were tested for *in vivo* activities, through comparison of their AUDPC values (Table 2). *Ap*LAO had no effects on disease progression in the tomato plants, with AUDPC of 93, similar to previously observed effects (Erjavec et al., 2016). On the other hand, the *I. geotropa* extracts, *Cg*LAO, the mycelium extract and *Cg*mycLAO all significantly delayed disease progression in the tomato plants (*p* < 0.05 or < 0.01; Figure 11). This indicated that the protein in the extracts from the fruiting bodies and the mycelia of *I. geotropa* that had antibacterial activity was the purified *Cg*LAO and *Cg*mycLAO. The same observations were made previously for a *I. geotropa* extract, with AUDPC of 75% (Erjavec et al., 2016). The AUDPC for the *Cg*mycLAO extract (75%) was the same as that for the *Cg*LAO fruiting body extract. The AUDPCs for *Cg*LAO and *Cg*mycLAO were higher compared to the extracts, at 79 and 85%, respectively.

**TABLE 2:** *In vitro* antibacterial activities against *R. solanacearum* and *in vivo* (tomato cv. “Moneymaker”) disease activities of the protein extracts and purified proteins.

	*In vitro* antibacterial activity				
Extract/purified protein fraction	vs. *R. solanacearum*		Pathogenicity (tomato cv. “Moneymaker”)		
	Level	Type	AUDPC^a^ (% positive control)		Significance vs. positive control^b^ (Yes/No)
			1	2	
*A. phalloides* fruiting body	+++	Bactericidal	114^c^	98^c^	No
*Ap*LAO	+++	Bactericidal	nt	93	No
*I. geotropa* fruiting body	+++	Bactericidal	76^c^	75^c^	Yes
*Cg*LAO	+++	Bactericidal	nt	79	Yes
*I. geotropa* mycelium	+++	Bactericidal	nt	75	Yes
*Cg*mycLAO	+++	Bactericidal	nt	85	Yes

*^*a*^AUDPC, area under the disease progress curve: quantitative summary of disease intensity over time, calculated for tomato plants infected with mixtures of *R. solanacearum* NIB Z 30 and protein samples. Symptom severity evaluated 14 days post-inoculation. AUDPC expressed relative to tomato positive control. ^b^Within groups, the time points at which the distribution of symptoms differs from the corresponding distribution in the positive control group, are marked by asterisk above columns in Figure 11 (chi-squared test; ^∗^*p* < 0.05; ^∗∗^*p* < 0.01). ^*c*^Published previously (Erjavec et al., 2016). +++, complete inhibition of bacteria: no growth observed (≤15% positive control A_595_). nt, not tested.*

### LAOs Have Antibacterial Activities Against Several Plant Pathogenic Bacteria

We assessed the scope of fungal LAOs antibacterial activities by analyzing their effect on the growth of different plant pathogenic bacteria. *Ap*LAO (33 μg/mL) and *Cg*LAO (103 μg/mL) showed inhibitory activities against several selected catalase-positive bacteria, with the exception of *Erwinia amylovora* (Table 3). Furthermore, *Ap*LAO (100 μg/mL) completely inhibited growth measured at 24h in YPG medium of the following bacteria: *A. tumefaciens* (NCPPB 2437), *Enterobacter* sp. (NCCPB 4168), *P. atrosepticum* (NIB Z 620), *P. carotovorum* (NIB Z 623), *D. chrysanthemi* (NCPPB 402), *E. coli* (GSPB 48), *R. mannitolilytica* (CFBP 6737), and *X. arboricola* pv. pruni (NCPPB 416). *Cg*mycLAO appeared to be less effective *in vitro* compared to *Ap*LAO and *Cg*LAO, as the similar activities were seen at higher *Cg*mycLAO concentrations.

**TABLE 3:** Inhibitory activities of *Ap*LAO, *Cg*LAO and *Cg*mycLAO against the selected bacteria.

Bacteria	Strain	Medium^a^	Growth after 24 h (% positive control)		
			*Ap*LAO	*Cg*LAO	*Cg*mycLAO
			33 μg/mL	103 μg/mL	2.1 mg/mL
*Ralstonia solanacearum*	NCPPB 4156	CPG	0	0	0
*Dickeya* sp.	NIB B16	CPG	77.7	0	0
*Dickeya* sp.	NIB S1	CPG	50.0	0	0
*Dickeya fangzhongdai*	DSMS 101947	CPG	278	0	0
*Erwinia amylovora*	NCPPB 683	KB	100	69.7	90.9
*Pseudomonas syringae pv. syringae*	NCPPB 281	KB	97.4	0	0
*Clavibacter michiganensis* subsp. *michiganensis*	NCPPB 2979	NBYE	0	0	0

*^*a*^composition of media as described in Schaad et al. (2001).*

## Discussion

Here, we have described the purification of antibacterial LAOs from two mushrooms: the poisonous death cap *A. phalloides* (*Ap*LAO) and the edible trooping funnel *I. geotropa* (*Cg*LAO). These were purified through two chromatographic steps following the initial activation and solubilization of the active LAO species using NaSCN and urea. More complex procedures have been used previously for the isolation of LAOs from fungal fruiting bodies and mycelia. Four- to six-step procedures comprising several steps of different types of chromatographies and protein precipitation were used for toxophallin and Trp-oxidase purification (Furuya et al., 2000; Stasyk et al., 2008).

In our initial purification and identification of the antibacterial component in crude extracts of *A. phalloides*, *I. geotropa*, and *I. geotropa* mycelia, in all cases the antibacterial activities following gel filtration ran close to the void volume of the column. This indicated a relatively high molecular mass species of around 200 to 300 kDa. This would be attributable to relatively large protein assemblies, which can result from aggregation with other proteins or from multimerization of LAOs in the mushroom tissues and the crude extracts. Another observation was that there was significant precipitation of bioactive material during the concentrating of the crude extracts. When dissolved in 3 M urea, these precipitates contained a significant amount of bioactive material. With the aim to help with the purification, the chaotropic agents NaSCN and urea were included in all of the crude extracts and gel filtration buffers with *I. geotropa*, as this provided an immediate several-fold increase in the measurable LAO activities in the *I. geotropa* extracts, and eventually in greater final yield of the corresponding LAO enzymes. This tendency to precipitate also remained during the next purification step of hydrophobic chromatography. A relatively low concentration of ammonium sulfate (0.85 M) had to be used to achieve binding of the sample to the phenyl-Sepharose, the use of higher concentrations resulted in increased precipitation of protein that showed antibacterial and LAO activities.

The molecular masses of the purified *Ap*LAO and *Cg*LAO were estimated as 72 and 66 kDa, respectively, from the SDS-PAGE analysis, and between 120 and 130 kDa by gel filtration using native conditions, which suggested that these enzymes were dimeric in nature. This is consistent with the molecular masses of LAOs isolated from fruiting bodies and mycelia of other basidiomycetes. Monomers of L-Trp-oxidase from *Coprinus* sp. (Furuya et al., 2000) and an LAO from *Hebeloma cylindrosporium* (*Hc*LAO) (Nuutinen et al., 2012) had molecular masses of 68 and 67 kDa, respectively, and assembled into hexamers (420 kDa) and dimers (140 kDa), respectively. Monomers of toxophallin (Stasyk et al., 2010) and toxovirin (Antonyuk et al., 2010) were identified as 55 kDa proteins. The differences in the previously reported molecular mass of toxophallin compared to that in the present study can be attributed to the low accuracy of molecular mass estimations with SDS-PAGE. A range of molecular masses of LAO monomeric subunits have been reported from various microbial and animal sources, which have ranged from 43 kDa for LAO from *Streptococcus oligofermentas* (Tong et al., 2008) to 85 kDa for LAO from sea hare *Aplysia kurodai* (Kamiya et al., 1986). The molecular masses of the newly identified fungal LAOs are within the prevalent range of LAO molecular masses between 60 and 70 kDa. With the exception of the monomeric LAO escapin from sea hare *Aplysia californica* (Yang et al., 2005), these enzymes are mostly active as oligomers. They have included mainly dimers (Geueke and Hummel, 2002; Kitani et al., 2007; Yang et al., 2011; Guo et al., 2012), but also trimers (Kitani et al., 2010), and tetramers (Kamiya et al., 1986), with molecular masses ranging from 100 to 340 kDa. The initial isolation procedures that have generally been adapted to allow for such high molecular mass protein assemblies have indicated the presence of an array of possible aggregates or multimeric proteins in the fruiting bodies that show strong antibacterial activities. These have then generally been dissociated using chaotropic ions and urea during the purification procedures, which has resulted in predominantly LAO dimers present under the conditions described.

The isoelectric points of LAOs can be relatively variable. While most are acidic proteins with pI between pH 4 and pH 5, a few have higher pIs, above pH 8 (Geueke and Hummel, 2002; Guo et al., 2012; Vargas et al., 2013). In contrast to the isoelectric points of *Ap*LAO and *Cg*LAO here as approximately at pH 6.5, slightly lower isoelectric points have been reported for toxophallin (pH 5.7) (Stasyk et al., 2008) and *Hc*LAO (pH 5.4) (Nuutinen et al., 2012).

N-glycosylation was shown here for *Ap*LAO, while *Cg*LAO did not appear to be N-glycosylated. The glycosylation status of the LAOs from basidiomycetes has not been investigated, however, an indication of glycosylation of *Hc*LAO was shown by 2D-PAGE analysis, which yielded several spots (Nuutinen et al., 2012). Animal and fungal LAOs with antibacterial activities have been shown to be glycoproteins (Yang et al., 2005, 2011; Kitani et al., 2007; Alves et al., 2008), although N-glycans appear not to be involved in the antibacterial activities, as deglycosylation did not reduce the antibacterial activity for a LAO from rockfish (Kitani et al., 2007), and a non-glycosylated bacterial LAO has shown antibacterial activity (Tong et al., 2008). Our results confirm that N-glycans are not essential for antibacterial activity of LAOs as both non N-glycosylated *Cg*LAO and N-glycosylated *Ap*LAO showed strong antibacterial activity.

The slightly acidic pH optimum between pH 5 and pH 6 seen here for *Ap*LAO and *Cg*LAO differs from more basic pH optima reported for L-Trp oxidase, at pH 7 (Furuya et al., 2000), and for LAOs from *Hebeloma* spp. and *Laccaria bicolor*, at pH 8 (Nuutinen and Timonen, 2008; Nuutinen et al., 2012). LAO activities that show a broad pH range are, however, common to basidiomycete LAOs, thus being similar to, although not as expanded as, *Ap*LAO and *Cg*LAO here. LAO activities with broad pH ranges have been reported, such as from pH 6 to pH 10 for *Hebeloma* LAOs, and from pH 5 to pH 11 for *Coprinus* L-Trp-oxidase (Furuya et al., 2000; Nuutinen and Timonen, 2008). Snake venom LAOs have their pH optimum between pH 7 and 8.5 (Tan and Fung, 2008), and microbial LAOs show a broader pH optimum usually between pH 6 and pH 9 (Geueke and Hummel, 2002; Kurosawa et al., 2009; Yang et al., 2011; Pollegioni et al., 2013). Here described fungal LAOs showed extraordinarily wide pH optimum, which strengthens their potential for a wide array of applications.

L-amino acid oxidases have shown either broad or very narrow substrate specificities. The broad substrate specificities reported here for *Ap*LAO and *Cg*LAO were similarly reported for *Hc*LAO and toxovirin (Antonyuk et al., 2010; Nuutinen et al., 2012), while L-Trp-oxidase from *Coprinus* has a narrow substrate specificity (Furuya et al., 2000). Converesely, snake venom LAOs generally have high specificities toward hydrophobic or aromatic amino acids, such as L-Phe, L-Leu, L-Met, and L-Ile (Lukasheva et al., 2011; Guo et al., 2012), and several marine animal LAOs have high specificities toward positively charged amino acids, such as L-Lys and L-Arg (Jimbo et al., 2003; Yang et al., 2005; Kitani et al., 2007, 2010). Broad substrate specificities for charged and aromatic amino acids have been shown for some bacterial (Geueke and Hummel, 2002; Tong et al., 2008) and fungal (Davis et al., 2005) LAOs, while others have shown very narrow specificities against one L-amino acid, and these have been named accordingly (Kusakabe et al., 1980; Arima et al., 2009; Pollegioni et al., 2013; Hossain et al., 2014).

*Amanita phalloides* and *Cg*LAO are particularly stable enzymes that are resistant to repeated freezing and thawing cycles (our observations). In this regard, fungal LAOs are more similar to microbial enzymes that tend to be more robust and stable compared to those from animals. With a few exceptions, snake venom LAOs are thermo-labile enzymes that are inactivated by freezing but remain stable at 4°C (Tan and Fung, 2008; Guo et al., 2012; Pollegioni et al., 2013).

*Amanita phalloides*, *Cg*LAO and *Cg*mycLAO all showed broad specificities for their antibacterial actions, as they showed activities against both Gram-negative and Gram-positive bacteria, albeit that these were less pronounced for the Gram-positive bacteria. Some degree of specificity was shown in the screening of their activities on the various plant pathogenic bacteria, where *Dickeya* spp. showed some resistance, and growth of *Erwinia amylovora* was not affected. This is consistent with other known LAOs, which in general have broad antibacterial activities that encompass both Gram-positive and Gram-negative bacteria, while their activities against fungi and yeast are generally lower (Yang et al., 2005; Guo et al., 2012).

The mechanism of the antibacterial activities here is attributed to the LAO enzymatic activity that leads to formation of the toxic H_2_O_2_. Therefore, their antimicrobial activities can be abolished by addition of catalase. The addition of catalase suppressed the antibacterial activities of *Ap*LAO, *Cg*LAO, and *Cg*mycLAO against *R. solanacearum*, *E. coli* and *L. lactis*, which confirmed that these antibacterial activities are the consequence of H_2_O_2_ formation. Furthermore, significant contributions to these LAO activities comes from depletion of substrate L-amino acids and accumulation of other intermediates and their final products. For example, production of ammonia leads to a less acidic medium, and α-keto acids can act as siderophores (Lukasheva et al., 2011; Hossain et al., 2014). In the present study, the addition of L-Leu to the minimal medium significantly enhanced the antibacterial activities of *Ap*LAO, *Cg*LAO, and *Cg*mycLAO by providing more substrate for the LAO enzymatic activity and boost the production of H_2_O_2_.

The effects of LAO activities against the different parameters of the bacterial growth curves were more pronounced in terms of the extended lag phase on Gram-positive *L. lactis*, while all three monitored growth parameters were affected in *E. coli*. These were most probably caused by the oxidative stress mediated by H_2_O_2_ as similar effects comprising a prolonged lag phase and lower growth rates upon exposure to different H_2_O_2_ levels have been shown for different *Aeromonas* and *Vibrio* spp. (Wang et al., 2004), as well as for *E. coli* (Sahu and Behuria, 2018).

Binding of LAOs to the bacterial surface has been shown, which presumably enhances their antibacterial activities by the consequent increased local concentrations of H_2_O_2_ (Ehara et al., 2002; Lee et al., 2011). Despite several attempts, we have not been able to conclusively confirm the binding of *Ap*LAO or *Cg*LAO to the surface of *R. solanacearum*.

Another aspect of these LAO activities is the morphological changes that were detected for *R. solanacearum* after the addition of *Cg*LAO here. These changes were mainly evident as the formation of bulges in the bacterial surface, and as a consequence, many bacteria became more curved than rod shaped. The *S. schlegeli* antibacterial protein and H_2_O_2_ have been shown to produce similar morphological changes to bacteria. However, these changes differ among the bacterial species, and can include bleb-like protrusions on the surface, cell elongation, and pore formation, which can be accompanied by the rough appearance of the cell surfaces (Kitani et al., 2008; Wang et al., 2011).

The morphological and ultrastructural changes in *R. solanacearum* were probably the consequences of the rearrangement of the PHA granules, which are believed to be involved in oxidative stress tolerance (Castro-Sowinski et al., 2010). This was also confirmed here by the increased fibrillated structure around the PHA granules. It has been shown that PHA synthesis enhances the tolerance of plant pathogenic *Pseudomonas* to oxidative stress (Fones and Preston, 2012). Reactive oxygen species, such as H_2_O_2_, can even stimulate PHA synthesis, as a protective function against stress. This is in part mediated by the protection of proteins from oxidative damage, with a protective efficiency greater than that of trehalose (Obruca et al., 2016; Al Rowaihi et al., 2018). The present study also indicated changes in the cell wall structure of *R. solanacearum* after the treatment with *Cg*LAO. Instead of the cell wall being visibly divided into the inner and outer membranes, with the *Cg*LAO treatment the majority of the cells had a less pronounced and structured cell wall. Interestingly, morphological changes to mammalian cells in culture have been reported after treatments with *Ap*LAO, which showed a periplasmic localization (Pišlar et al., 2016).

The pathogenicity tests in the present study confirm that the *I. geotropa* LAO isolated from the fruiting bodies and mycelia can delay disease progression in tomato plants. To the best of our knowledge, this is the only study that has repeated *in vivo* testing and confirmed previously published results (Erjavec et al., 2016). Furthermore, in addition to the antibacterial activities defined for the extracts, these antibacterial activities were substantiated *in vivo* using the proteins purified from the extracts. The effect of the *Cg*LAO fruiting body extract was stronger compared to the purified proteins *Cg*LAO and *Cg*mycLAO. This indicated that these enzymes might interact with other proteins, peptides or compounds in the extracts that enhanced their antibacterial activities but were lost during the purification. Interestingly, the antibacterial activities of *Ap*LAO appeared to be greater *in vitro*, while the pathogenicity tests showed it as ineffective *in vivo*. Although *Ap*LAO and *Cg*LAO are both LAOs and they show similar biochemical characteristics, their LAO enzymatic activities do not appear to be sufficient to provide antibacterial protection to these plants *in vivo*. Potentially, the glycosylation state or interactions with other proteins confer this specificity and enhanced activity. The stronger activity of *Cg*LAO in terms of the prolongation of the lag phase might have provided the plant with more time to mount an antibacterial response. On the other hand, the different *in vivo* activities of *Ap*LAO and *Cg*LAO might be the consequence of their interactions with the plant defense system. Importantly, the protective activity of *Cg*LAO purified from the fruiting bodies was mirrored by *Cg*mycLAO purified from the mycelia, as well by the simple aqueous extracts of the cultured mycelia, thus indicating their potential for their use as plant protection agents.

## Conclusion

We identified new fungal LAOs with antibacterial activity and described the process of their purification from higher fungi as well as their comprehensive biochemical characterization. We showed their antibacterial activity *in vitro* against a broad range of Gram-negative and Gram-positive bacteria encompassing several species of phytopathogens. Moreover, *in vivo* antibacterial activity was demonstrated for LAOs from *I. geotropa* fruiting bodies and mycelia in tomato plants, while the strong antibacterial effect of *Ap*LAO *in vitro* had no effect on disease progression *in planta*. This raises an important point to test the antibacterial effect of new candidate phytoprotective agents observed *in vitro*, also *in vivo* in the early steps of their development. We have also demonstrated that antibacterial and LAO activity is present and expressed in cultured mycelia of *I. geotropa*, which indicates that a constant source is available and strengthens their potential to be used as new biological phytoprotective agents. Similar antibacterial activity based on LAOs was observed in other fungal species showing fruiting bodies of higher fungi to be a valuable source of antimicrobials.

## Data Availability Statement

All datasets generated for this study are included in the article/Supplementary Material.

## Author Contributions

JB designed the study and purified proteins. JS designed and performed biochemical experiments and those using model microorganisms. JE and TD designed and performed experiments with *R. solanacearum.* MT performed and analyzed TEM. MR and JK provided the resources. JB and JS wrote the manuscript. JE, MT, MR, and JK reviewed and edited the manuscript.

## Conflict of Interest

The authors declare that the research was conducted in the absence of any commercial or financial relationships that could be construed as a potential conflict of interest.

## Footnotes

1 http://www.indexfungorum.org

## Abbreviations

*Ap*LAO: *Amanita phalloides* L-amino acid oxidase
AUDPC: area under the disease progress curve
*Cg*LAO: *Infundibulicybe* (previously *Clitocybe) geotropa* L-amino acid oxidase
*Cg*mycLAO: mycelium-derived *Infundibulicybe* (previously *Clitocybe) geotropa* L-amino acid oxidase
CSM: complete supplement mixture
*Hc*LAO: *Hebeloma cylindrosporium* L-amino acid oxidase
LAO: L-amino acid oxidase
PBS: phosphate-buffered saline
PHA: polyhydroxyalkanoate
YPG: yeast peptone glucose.
