# Supplementary material for: L-Amino Acid Oxidases From Mushrooms Show Antibacterial Activity Against the Phytopathogen Ralstonia solanacearum
Source: Front Microbiol. 2020 May 19;11:977. doi: 10.3389/fmicb.2020.00977 (PMC7248570; doi:10.3389/fmicb.2020.00977)
Supplement: Supplementary file 1 [file Data_Sheet_1.pdf]

Supplementary Material

1 Supplementary Figure S1

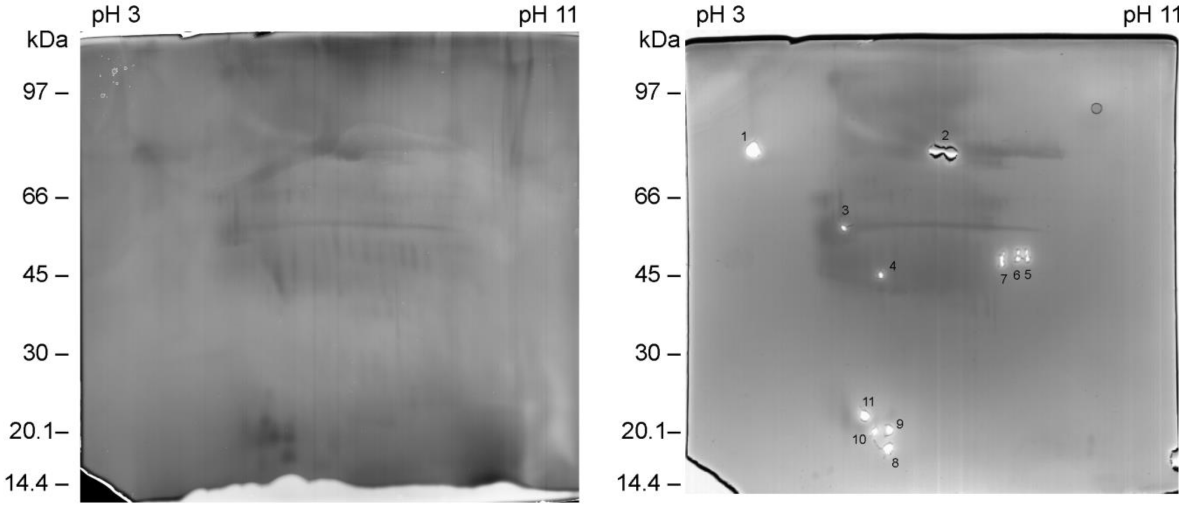

Supplementary Figure S1. 2D-PAGE analysis of the 180 kDa protein complex with antibacterial activity from *I. geotropa*. After preparative Native PAGE of the gel filtration fraction exhibiting antibacterial activity the prominent band at 180 kDa was excised, eluted overnight by diffusion from gel pieces and analysed by 2D-PAGE. After silver staining (left panel) spots were excised (right panel) and following in-gel trypsin digestion analysed by peptide mass fingerprinting using ion trap mass spectrometer HPLC-Chip-LC/MSD Trap XCD Ultra and Mascot in-house server using MS/MS Ion Search. However, no significant hits were retrieved from databases.

2 Supplementary Figure S2

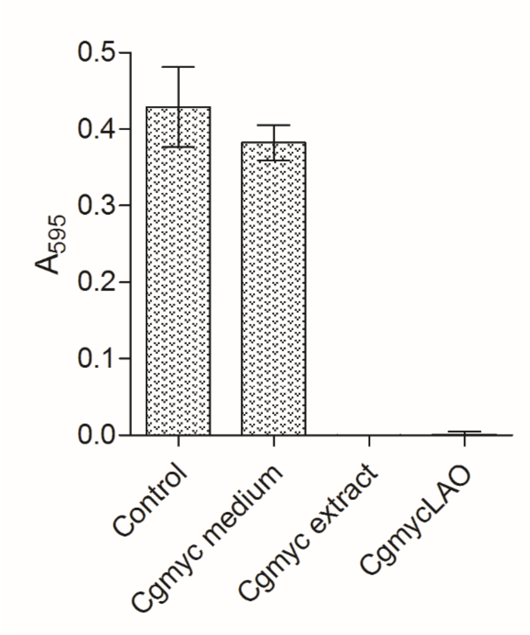

Supplementary Figure S2. Analysis of antibacterial activity against *R. solanacearum* of *I. geotropa* mycelium extract, sucrose malt yeast extract medium in which it was grown and gel filtration fraction exhibiting LAO activity. Average A_{595} values after 24 h and standard deviation of three technical replicates are represented.

3 Supplementary Figure S3

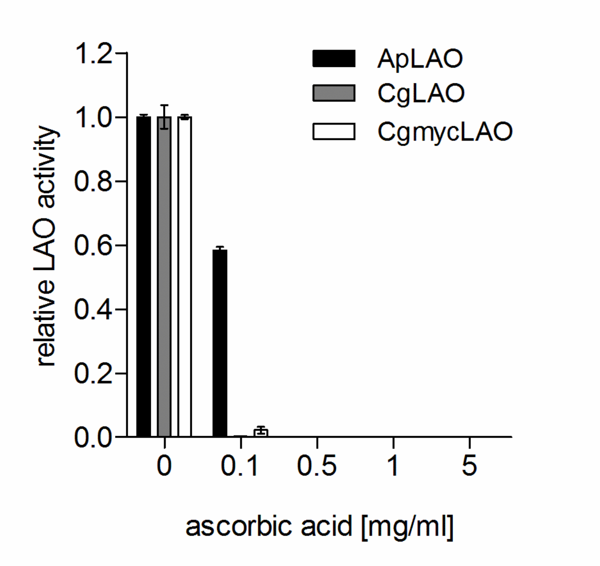

Supplementary Figure S3. Inhibition of LAO activity by ascorbic acid. LAO activity was measured in the presence of different concentrations of ascorbic acid at pH 5.5 using L-Leu as substrate. Mean with SD is shown.

4 Supplementary Figure S4

Supplementary Figure S4. Comparison of LAO and antibacterial activities in gel filtration fractions. Extracts of fruiting bodies of 15 mushroom species including 14 basidiomycetes and 1 ascomycete (Table 1) were fractionated by gel filtration chromatography using Sephacryl S300. Absorbance at 280 nm (white circles) was measured in fractions. Antibacterial activity of fractions was determined and is expressed as percent growth of *R. solanacearum* relative to positive control (PC) measured as absorbance at 595 nm after 24 h incubation (bold dashed line). LAO activity using 5 mM L-Leu (red circles) or 0.1 % CSM (blue triangles) as substrate at pH 5.5 was measured spectrophotometrically and using 0.1 % CSM as substrate for in-gel detection (insets shown only for species and fractions in which LAO activity was detected, those not shown had no in-gel activity).

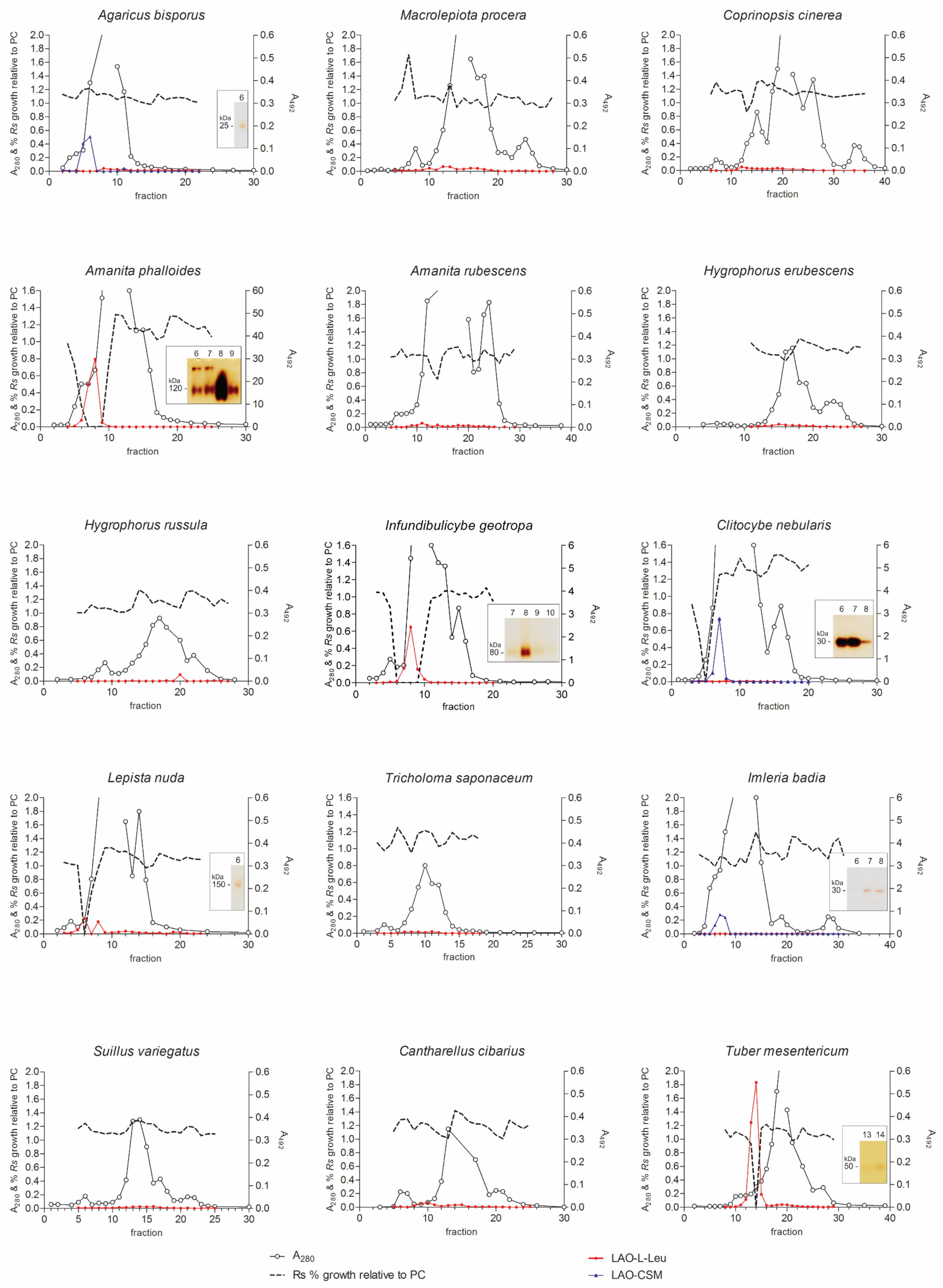
Agaricus bisporus
Macrolepiota procera
Coprinopsis cinerea
Amanita phalloides
Amanita rubescens
Hygrophorus erubescens
Hygrophorus russula
Infundibulicybe geotropa
Clitocybe nebularis
Lepista nuda
Tricholoma saponaceum
Imleria badia
Suillus variegatus
Cantharellus cibarius
Tuber mesentericum
A280 & % Rs growth relative to PC
A492
fraction
kDa
A280
Rs % growth relative to PC
LAO-L-Leu
LAO-CSM

5 Supplementary Figure S5

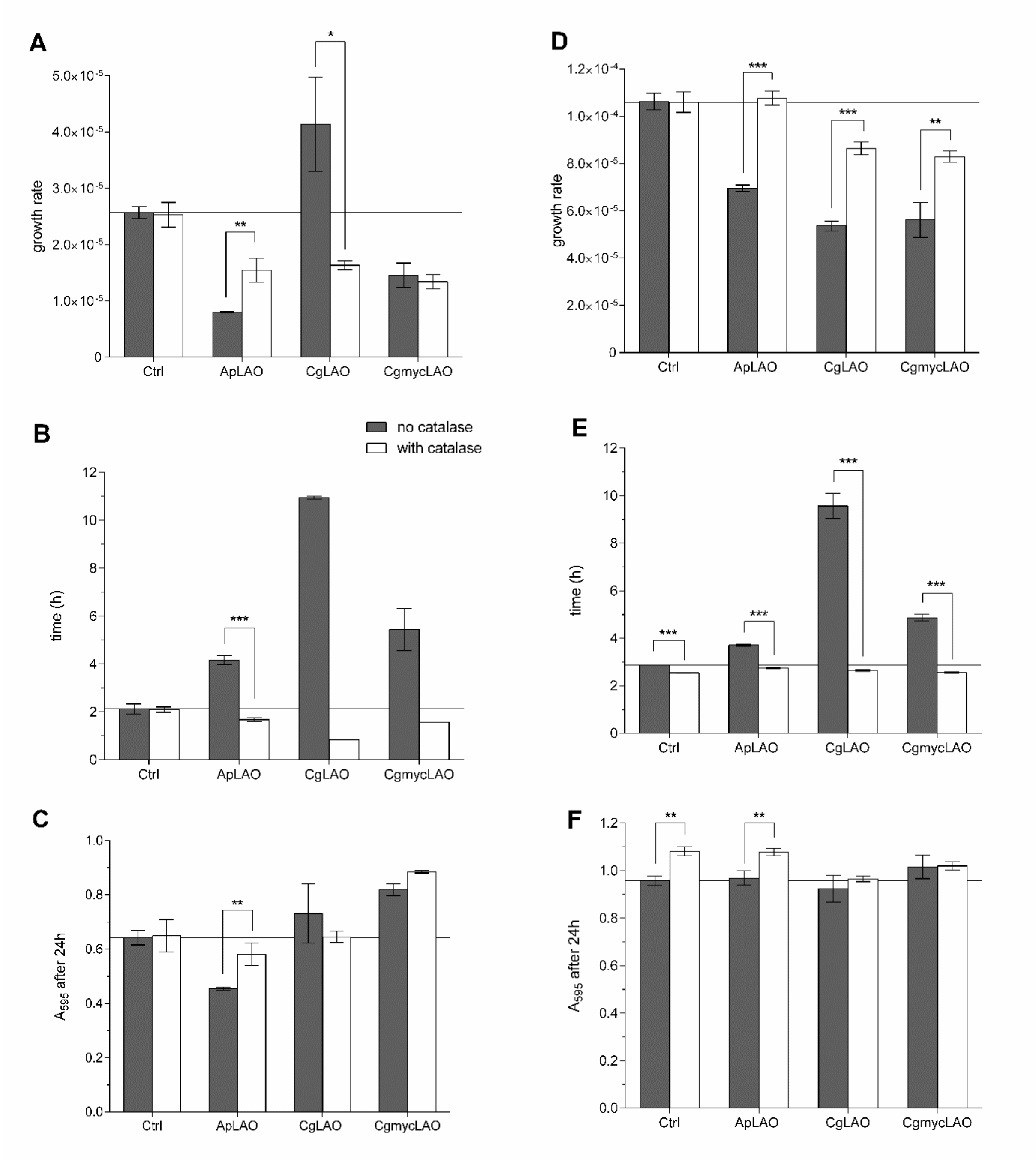

Supplementary Figure S5. The antibacterial effect of LAO activity is counteracted by the addition of catalase (1000 U/ml). Growth rates, lag phase length and absorbance after 24 h are shown for *E. coli* (panels A, B, C) and *L. lactis* (panels D, E, F) in the presence of ApLAO (10.7 µg/ml), CgLAO (11.6 µg/ml for *E. coli* and 33 µg/ml for *L. lactis*), and CgmycLAO (86 µg/ml). The legend in panel B applies to all panels. Mean with SD is shown with aterisks indicating statistical significance as determined by Students's ttest compared to control without catalase: * $p < 0.05$, ** $p < 0.01$, *** $p < 0.001$.

6 Supplementary Figure S6

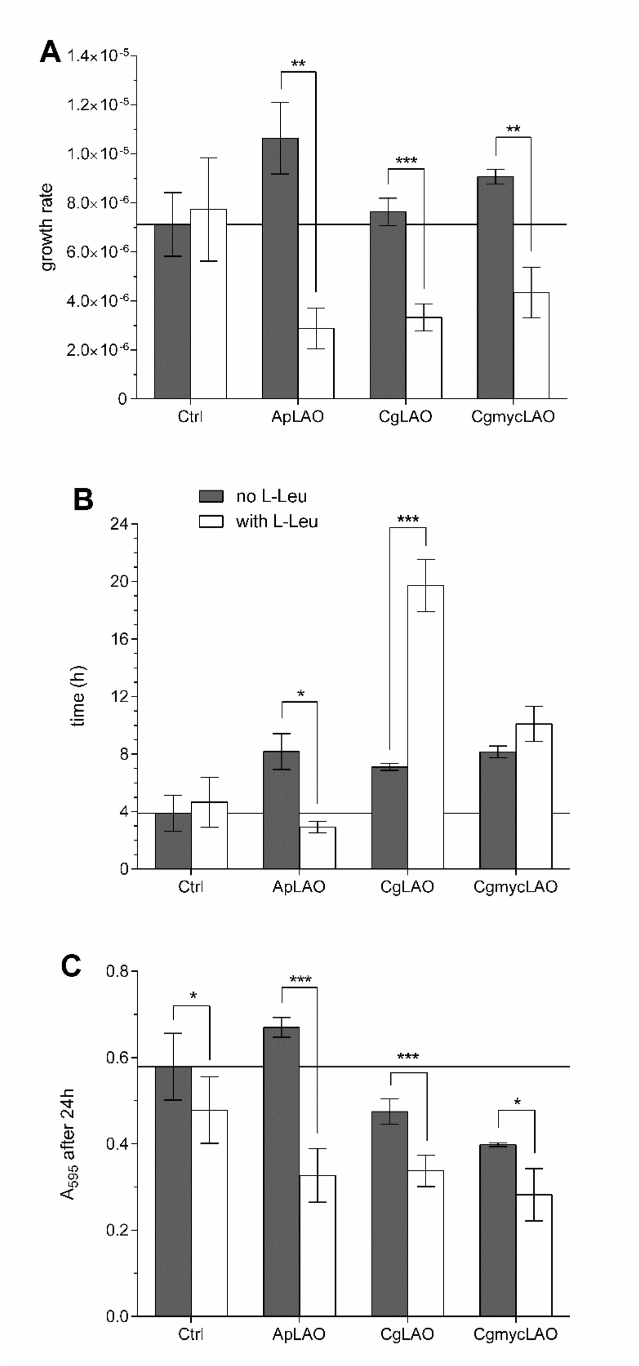

Supplementary Figure S6. The antibacterial effect of LAO activity against *E. coli* is exacerbated by the addition of L-Leu (5 mM) in minimal medium. Growth curves were followed for 24 h at 30°C and analysed using DMFit. Growth rate (A), lag phase length (B) and absorbance after 24 h (C) are shown in the presence of ApLAO (10.7 µg/ml), CgLAO (33 µg/ml), and CgmycLAO (86 µg/ml). The legend in panel B applies to all panels. Mean with SD is shown with aterisks indicating statistical significance as determined by Students's ttest compared to control without L-Leu addition: * $p < 0.05$, ** $p < 0.01$, *** $p < 0.001$.
